# Pyrazole derivatives of pyridine and naphthyridine as proapoptotic agents in cervical and breast cancer cells

**DOI:** 10.1038/s41598-023-32489-5

**Published:** 2023-04-01

**Authors:** Rima D. Alharthy, Faisal Rashid, Abida Ashraf, Zahid Shafiq, Steven Ford, Mariya al-Rashida, Muhammad Yaqub, Jamshed Iqbal

**Affiliations:** 1 Chemistry Department, Faculty of Science and Arts, King Abdulaziz University, Rabigh, 21911 Saudi Arabia; 2 Centre for Advanced Drug Research, COMSATS University Islamabad, Abbottabad Campus, Abbottabad, 22060 Pakistan; 3 Department of Chemistry, Kutchery Campus, The Women University Multan, Multan, 60000 Pakistan; 4 Institute of Chemical Sciences, Bahauddin Zakariya University, Multan, Pakistan; 5 Department of Pharmaceutical and Medicinal Chemistry, University of Bonn, An der Immenburg 4, 53121 Bonn, Germany; 6 Department of Pharmaceutical Sciences, Institute of Pharmacy and Biomedical Sciences, University of Strathclyde, Glasgow, UK; 7 Department of Chemistry, Forman Christian College (A Chartered University), Lahore, Pakistan

**Keywords:** Biochemistry, Chemistry

## Abstract

Cancer is one of the leading causes of death worldwide. The increasing prevalence and resistance to chemotherapy is responsible for driving the search of novel molecules to combat this disease. In search of novel compounds with pro-apoptotic potential, pyrazolo-pyridine and pyrazolo-naphthyridine derivatives were investigated against cervical cancer (HeLa) and breast cancer (MCF-7) cells. The anti-proliferative activity was determined through the MTT assay. Potent compounds were then analyzed for their cytotoxic and apoptotic activity through a lactate dehydrogenase assay and fluorescence microscopy after propidium iodide and DAPI staining. Flow cytometry was used to determine cell cycle arrest in treated cells and pro-apoptotic effect was verified through measurement of mitochondrial membrane potential and activation of caspases. Compounds **5j** and **5k** were found to be most active against HeLa and MCF-7 cells, respectively. G0/G1 cell cycle arrest was observed in treated cancer cells. Morphological features of apoptosis were also confirmed, and an increased oxidative stress indicated the involvement of reactive oxygen species in apoptosis. The compound-DNA interaction studies demonstrated an intercalative mode of binding and the comet assay confirmed the DNA damaging effects. Finally, potent compounds demonstrated a decrease in mitochondrial membrane potential and increased levels of activated caspase-9 and -3/7 confirmed the induction of apoptosis in treated HeLa and MCF-7 cells. The present work concludes that the active compounds **5j** and **5k** may be used as lead candidates for the development of lead drug molecules against cervical and breast cancer.

## Introduction

Cancer is a multi-factorial disease which is characterized by unchecked and abnormal cellular division of poorly differentiated cells. Tumorigenesis is a complex multistage process in which a group of cells, in a specific part of the body, starts unlimited replication due to genetic and epigenetic modifications^1,2^. Tumorigenesis involves an initiation step in which mutations in a cell population drive the cells to unchecked replication by by-passing apoptotic signals. Heterogeneity is observed in such cell clusters. Such cells produce larger populations, start affecting nearby cells, enlarge their size and consequently invade to other body parts or systems, a characteristic of malignancy known as metastasis^3^. Benign tumors are devoid of invasive nature due to sac like covering around them produced by the body’s defense mechanisms and they remain non-invasive unless a major mutational change induces malignancy. Benign tumors are easy to handle and can be effectively treated through conventional therapy, however, malignancy is life threatening. If a tumor is metastatic, it requires rigorous treatment, intensive care and critical monitoring of untoward effects^4^.

Cancer can be life threatening if not diagnosed and treated in the earlier stages. Oncological treatments include surgical removal of the developed cancer lump, radiation therapy, hormonal starvation therapy for certain cancers, immunotherapy by sensitizing cancer cells to the immune system and boosting its efficiency, stem cell transplants specially for non-solid tumors, genetic sequencing for identifying the genetic causes of a cancer and by chemotherapeutics to induce apoptosis in cancer cells^5^.

Dealing with multi-factorial and complex diseases is a big challenge and polychemotherapy or polypharmacy is facing problems: specifically patient non-compliance and resistance to chemotherapy. In synthetic pharmaceutical chemistry, “multi-target directed ligand” approach is an emerging strategy in which two or more pluripotent pharmacophores are hybridized into a single molecule to augment a therapeutic effect through multiple actions on a target^6–8^. Molecular hybridization is very promising in minimizing side effects^9^, facilitating access to target organs^10^ and making hybrid molecules resistant to resistance development^11–13^. Consequently, hybrid-drug designing is making an overall improvement in current therapeutics regarding efficacy, selectivity and safety.

Hybridizing two or more active pharmacophores to achieve better outcomes in disease management is now a regular practice. Pyrazolo-pyridine derivatives have been shown to be antimalarial, antiviral, anti-leishmanial, anti-tubercular, anti-leukemic, antifungal, anxiolytic, osteogenic and have, antitumor activity. Moreover, they have also been studied in management of drug and alcohol addiction, Alzheimer’s disease, infertility and gastrointestinal diseases^14^. Mohamed et al.^15^ synthesized pyrazolo-cyanopyridine derivatives having antitumor activity. Pyrazolo pyrimidine and pyridine derivatives were synthesized and tested against human laryngeal epidermoid carcinoma (HEp2) cells^16^. Two of the investigated compounds showed good antitumor activity. The compounds were also investigated on four cancer cell lines and all except one, were found to be potent against breast, hepatic, prostate and colon cancer cells^17^. Some novel pyrazolo pyridine derivatives were investigated for their anticancer activity in liver, colon and breast carcinoma cells, and all compounds were found to be antiproliferative agents^18^. Pyrazolopyridine derivatives also depicted good anticancer potential^19^. Thienopyridine and pyrazolopyridine derivatives showed good anti-proliferative activity in colon, liver and breast cancer cells^20^.

Naphthyridine derivatives have been reported as antifungal^21^, anticancer^22^, antibacterial^23^, GSK-3 inhibitors^24^, trypanocidal^25^, insecticidal^26^ acetyl and butyryl-cholinestrase inhibitors^27,28^. Their anticancer effect was previously studied by various research groups. A number of pyrazolo-naphthyridin derivatives showed noteworthy antiproliferative activity^29^. Similarly 1,8-naphthyridine derivatives, also showed good antitumor activity with IC_50_ values ranging from 1.47 to 35.3 µM in breast cancer cells^30^. These studies prompted us to investigate pyrazolo-pyridine (**4a**–**4n**) and pyrazolo-naphthyridine (**5a**–**5n**) for their potential to be used as antiproliferative agents.

## Results

### Anti-proliferative activity and structure activity relationship (SAR)

In search of new anti-neoplastic agents, the anti-proliferative potential of pyrazolopyridines (**4a**–**4n**) and pyrazolonaphthyridines (**5a**–**5n**) against MCF-7 and HeLa cells was evaluated by testing each compound at 100 µM. Non-cancerous BHK-21 cells were used to evaluate cytotoxicity of these compounds. Anti-proliferative potential was calculated as percentage of inhibition (% inhibition) and are shown in Table 1. The IC_50_ values were calculated and shown in Table 2. Compounds **5j** and **5k** were found to be the most active compounds with IC_50_ values of 6.4 ± 0.45 and 2.03 ± 0.23 µM against HeLa and MCF-7 cells respectively. Among twenty eight tested compounds, fourteen were found active against HeLa and MCF-7 cells. Compound **5j** was the most active against HeLa, whereas, then compound **5k** was the most active against MCF-7 cells (see Table 2).

**Table 1:** Anti-proliferative activity of pyrazolopyridine and pyrazolonaphthyridine derivatives against HeLa and MCF-7 cells.


Code	R	R_1_	HeLa cell line	MCF-7 cell line
			%Inhibition at 100 µM	
**4a**	H	H	34.53	28.17
**4b**	CH_3_	H	45.87	1.86
**4c**	F	H	57.80	26.82
**4d**	Cl	H	99.45	18.88
**4e**	Br	H	24.91	7.24
**4f**	NO_2_	H	26.17	5.23
**4g**	OCF_3_	H	38.64	15.23
**4h**	H	CH_3_	5.08	2.38
**4i**	CH_3_	CH_3_	39.67	1.42
**4j**	F	CH_3_	5.68	2.22
**4k**	Cl	CH_3_	3.84	11.90
**4l**	Br	CH_3_	39.31	1.78
**4m**	NO_2_	CH_3_	22.67	23.33
**4n**	OCF_3_	CH_3_	27.81	0.15
**5a**	H	H	97.91	46.36
**5b**	CH_3_	H	98.40	96.36
**5c**	F	H	69.25	4.12
**5d**	Cl	H	71.40	27.27
**5e**	Br	H	98.30	96.81
**5f**	NO_2_	H	26.99	9.54
**5g**	OCF_3_	H	61.30	31.36
**5h**	H	CH_3_	93.60	37.83
**5i**	CH_3_	CH_3_	63.50	58.18
**5j**	F	CH_3_	94.80	47.83
**5k**	Cl	CH_3_	96.40	69.50
**5l**	Br	CH_3_	81.60	28.18
**5m**	NO_2_	CH_3_	44.91	10.33
**5n**	OCF_3_	CH_3_	63.16	10.47
Cisplatin	–	–	98.63	73.12

**Table 2:** IC_50_ values of the most active pyrazolo-pyridine (**4a–4n**) and pyrazolo-naphthyridine (**5a–5n**) derivatives.

Code	HeLa Cell Line	MCF-7 cell line	BHK-21 cell line
	IC_50_ (µM) ± SEM		
**4c**	90.21 ± 2.18	–	49.21 ± 2.01
**4d**	51.63 ± 0.99	–	246.41 ± 6.71
**5a**	58.91 ± 0.39	–	177.42 ± 6.11
**5b**	32.12 ± 1.14	41.35 ± 0.47	126.23 ± 1.63
**5c**	10.31 ± 0.51	–	42.41 ± 1.95
**5d**	40.72 ± 1.42	–	166.81 ± 0.95
**5e**	30.23 ± 1.15	18.61 ± 0.29	84.11 ± 3.31
**5f**	69.11 ± 0.99	–	100.83 ± 2.61
**5h**	14.42 ± 1.21	–	31.42 ± 1.33
**5i**	61.93 ± 1.76	54.68 ± 0.63	133.91 ± 1.71
**5j**	6.43 ± 0.45	–	27.51 ± 1.32
**5k**	7.25 ± 0.12	2.03 ± 0.23	8.21 ± 0.57
**5l**	38.51 ± 0.63	–	151.62 ± 2.61
**5n**	32.73 ± 2.16	–	46.61 ± 1.03
Cisplatin	11.33 ± 0.78	6.22 ± 0.72	24.69 ± 0.37

*SEM* standard error of mean.

### Cytotoxic activity

**Figure 1:**
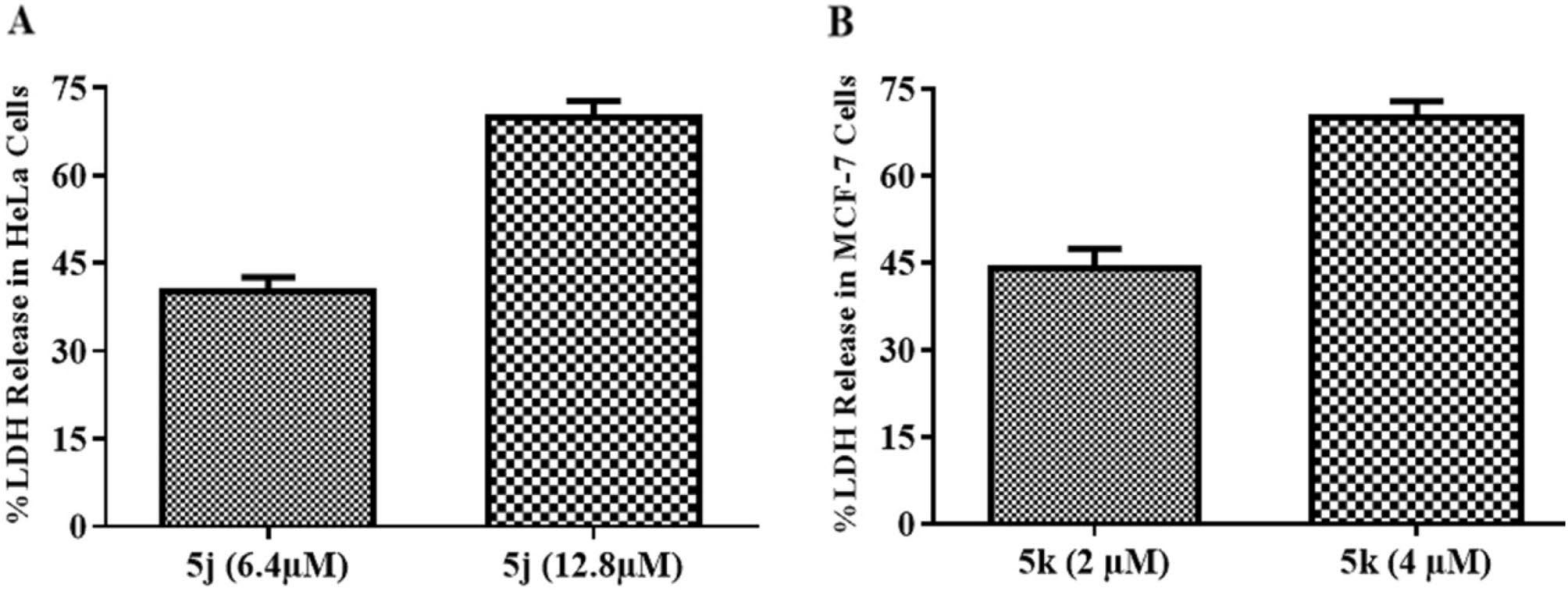
Cytotoxic effect of compounds **5j** and **5k** observed in (**A**) HeLa and (**B**) MCF-7 cells respectively by estimation of released LDH. Error bars represent standard deviation of three replicates.

The cytotoxic activity of active compounds **5j** and **5k** was determined by estimation of cytosolic lactate dehydrogenase (LDH) enzyme. A dose dependent increase in leaked LDH was measured in HeLa and MCF-7 cells after treatment with compounds **5j** and **5k** (Fig. 1) which showed good cytotoxic potential of potent compounds in these cells.

### Cytotoxicity, apoptosis and ROS determination by fluorescence microscopy

**Figure 2:**
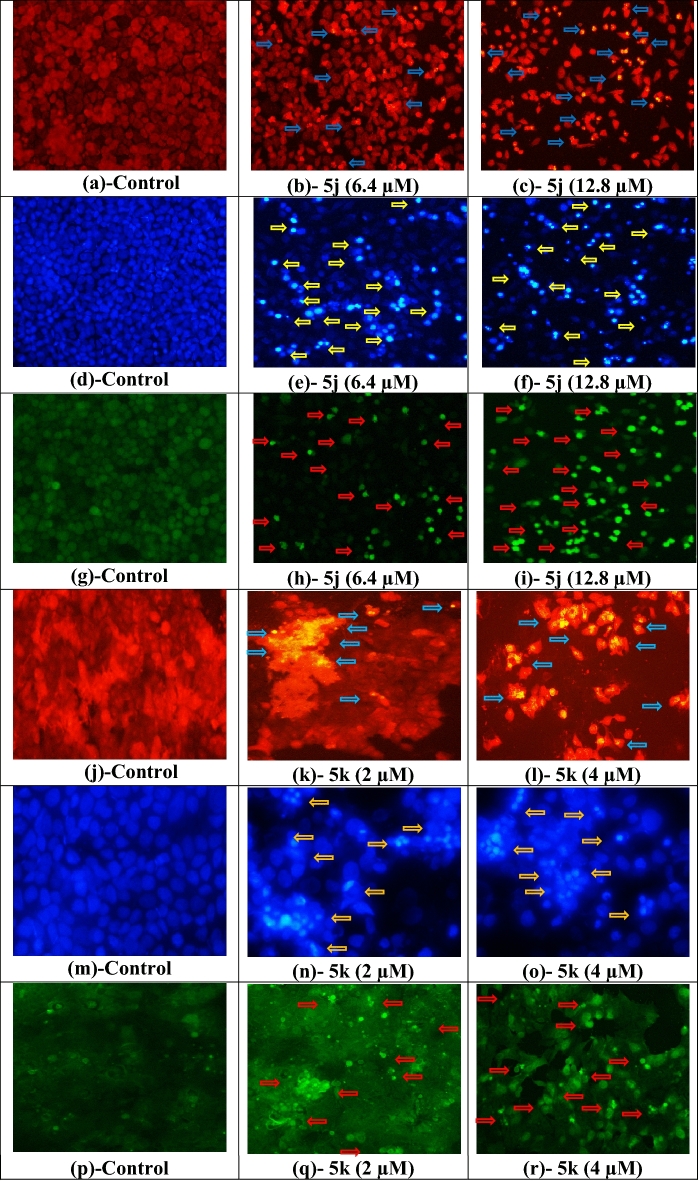
Fluorescence photomicrographs of untreated, compound **5j** treated HeLa cells after PI (**a**–**c**), DAPI (**d**–**f**) and H_2_DCF-DA (**g**–**i**) staining and compound **5k** treated MCF-7 cells after PI (**j**–**l**), DAPI (m,n,o) and H_2_DCF-DA (**p**–**r**) staining. Images are showing dose dependent cytotoxic, apoptotic and ROS generation indicated by arrows in compound **5j** treated HeLa and **5k** treated MCF-7 cells as compared to control.

Nuclear staining dyes propidium iodide (PI) and 4′,6-diamidino-2-phenylindole (DAPI) were used to identify cytotoxic and apoptotic effect of the most active compound **5j** and **5k**. Cell population decreased as dead cells became non-adherent while some apoptotic cells remained attached and were stained by PI and DAPI. Cell detachment, shrinkage of cells and condensation of nuclear material was observed [Fig. 2 (**a**–**f**, **j**–**o**)] in a dose dependent manner after treatment with compound **5j** and **5k**.

The production of ROS in treated cells was estimated by 2',7'-dichlorodihydrofluorescein diacetate (H_2_DCF-DA) staining which showed an intense green fluorescence in treated cells as compared to control class as shown in Fig. 2 (**g**–**i, p**–**r**).

### Analysis of cell cycle by flow cytometry

**Figure 3:**
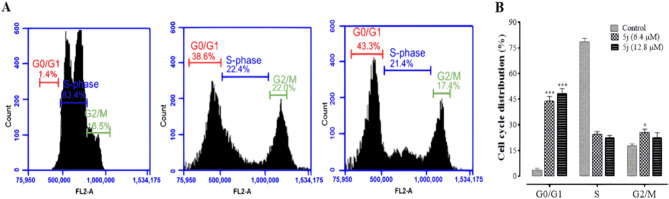
Compound 5j induced cell cycle arrest. (**A**) The representative results of cell cycle analysis obtained by flow cytometry in untreated (control) and compound **5j** treated cells. (**B**) The proportions of G0/G1, S and G2/M phases are represented as mean ± SD. *p ˂ 0.05, **p ˂ 0.01, ***p ˂ 0.001 versus control group (n = 3).

The DNA content measurement in each phase of cell cycle was evaluated through flow cytometry after PI staining of compound **5j** treated cells and compared with untreated control cells. Compound **5j** showed G0/G1 arrest in HeLa cells (Fig. 3).

### Determination of compound-DNA interactions

**Figure 4:**
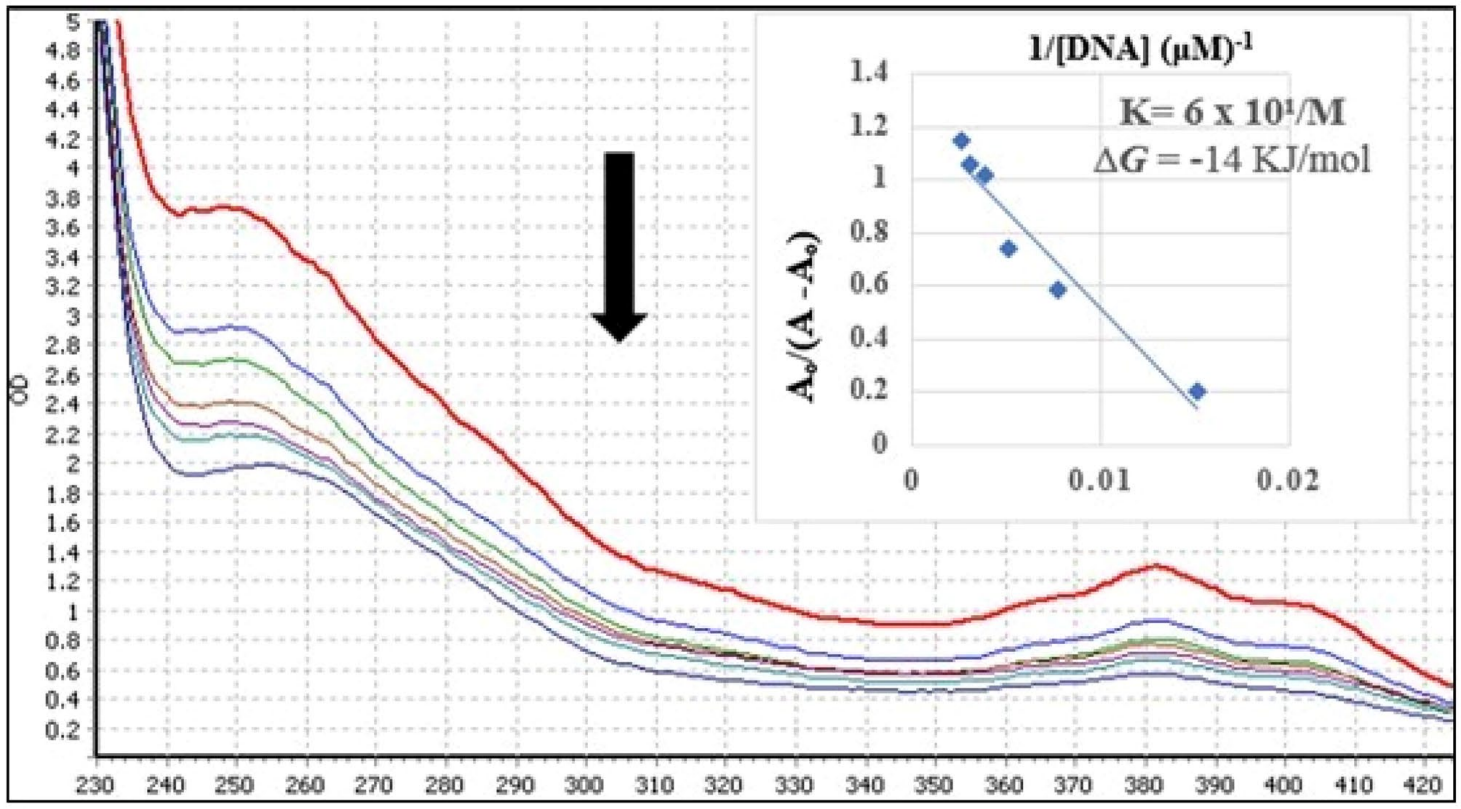
Absorption spectra of 200 μM of **5j** in absence (0 μM) and presence (66, 132, 198, 264, 330 and 396 μM) of DNA. The arrow direction shows increasing concentration of DNA. The graph is the plot of A°/(A-A°) versus 1/[DNA] for the determination of binding constant and Gibb’s free energy of **5j-DNA** product.

**Figure 5:**
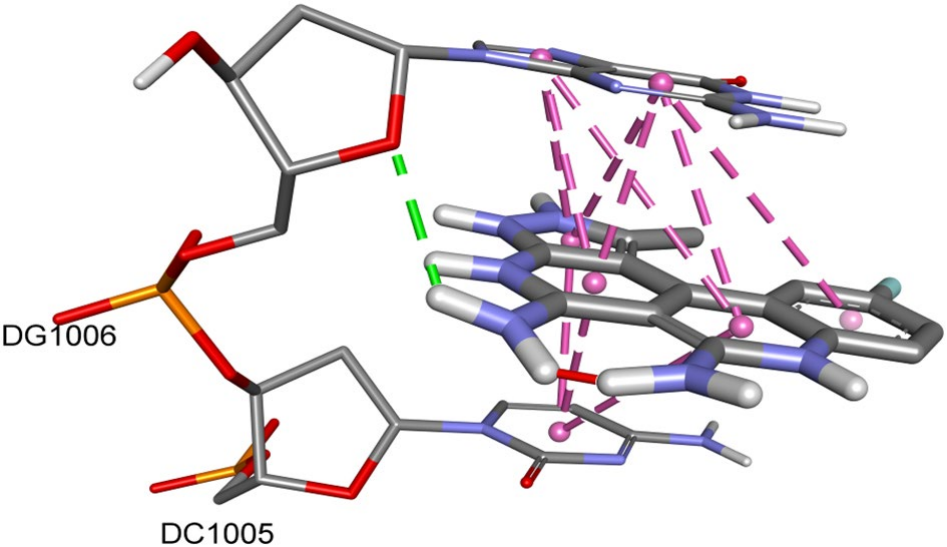
Docked conformation of DNA, compound **5j**.

The interaction of potent compound **5j** with DNA was studied: hypochromicity for two chromophores was observed with no red or blue shift, which indicated that compound **5j** was non-covalently bound to DNA. A Gibbs free energy of ΔG = − 14 kJ/moL was calculated for this reaction. Absorption spectra has been shown in Fig. 4. The compound-DNA interaction was further verified by in silico studies. Figure 5 shows the docked conformation of most active compound **5j** against DNA, exhibiting an intercalator binding mode (as expected due to the rigid planar rings of the molecule).

### Determination of DNA damage by comet assay

**Figure 6:**
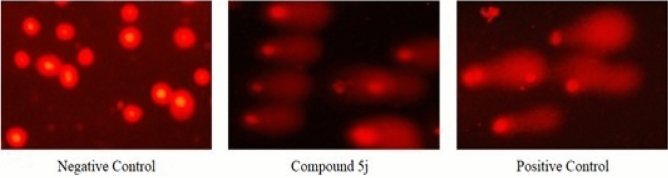
Representative images of comets obtained from untreated, compound **5j** treated and H_2_O_2_ treated HeLa cells by alkaline comet assay to assess DNA damage.

**Figure 7:**
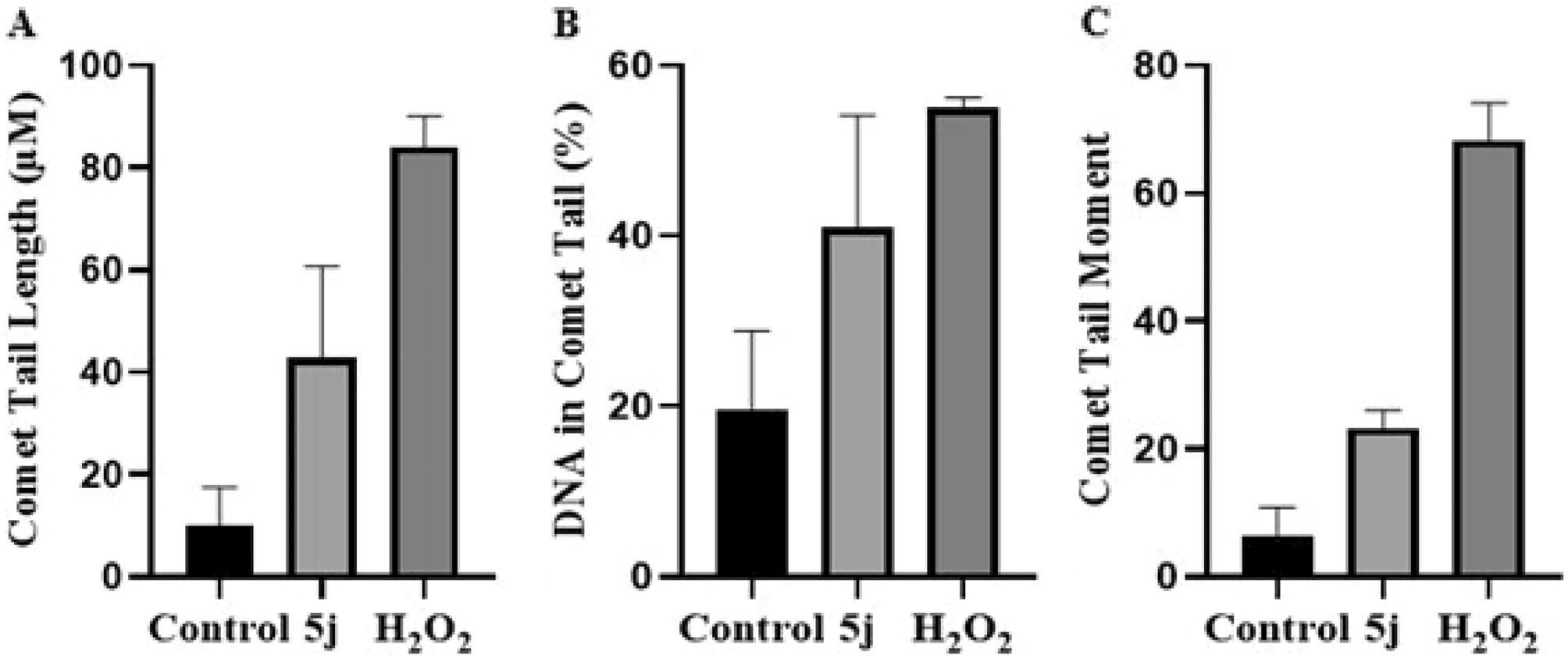
Quantitative analysis of (**A**) comet tail length, (**B**) percent DNA in tail and (**C**) comet tail moment in untreated, compound **5j** treated and H_2_O_2_ treated cells. Data are represented as mean ± SD in replicate of three. *p ˂ 0.05, **p ˂ 0.01, ***p ˂ 0.001 versus control group (n = 3).

The cervical cancer (HeLa) cells were treated with 6.4 µM of compound **5j**. Cells treated for 30 min with 20 µM of freshly prepared H_2_O_2_ were used as positive control. Images were captured as shown in Fig. 6 and analyzed by ImageJ and OpenComet. DNA damage was assessed by calculating the comet parameters tail length, %DNA in tail and tail moment as shown in Table 3 and Fig. 7. Compound **5j** showed DNA damage in treated cells, comparable to positive control.

**Table 3:** DNA damage parameters calculated for compound **5j,** untreated and H_2_O_2_ treated cells.

Sample	Tail length ± SD (µM)	%DNA in tail ± SD	Tail moment ± SD
**5j**	43.01 ± 10.27	41.02 ± 7.61	23.22 ± 1.61
Untreated	9.91 ± 4.36	19.65 ± 5.33	6.47 ± 2.51
H_2_O_2_	84.14 ± 3.43	55.11 ± 0.68	3.37

*SD* standard deviation.

### Measurement of mitochondrial membrane potential (ΔΨm)

**Figure 8:**
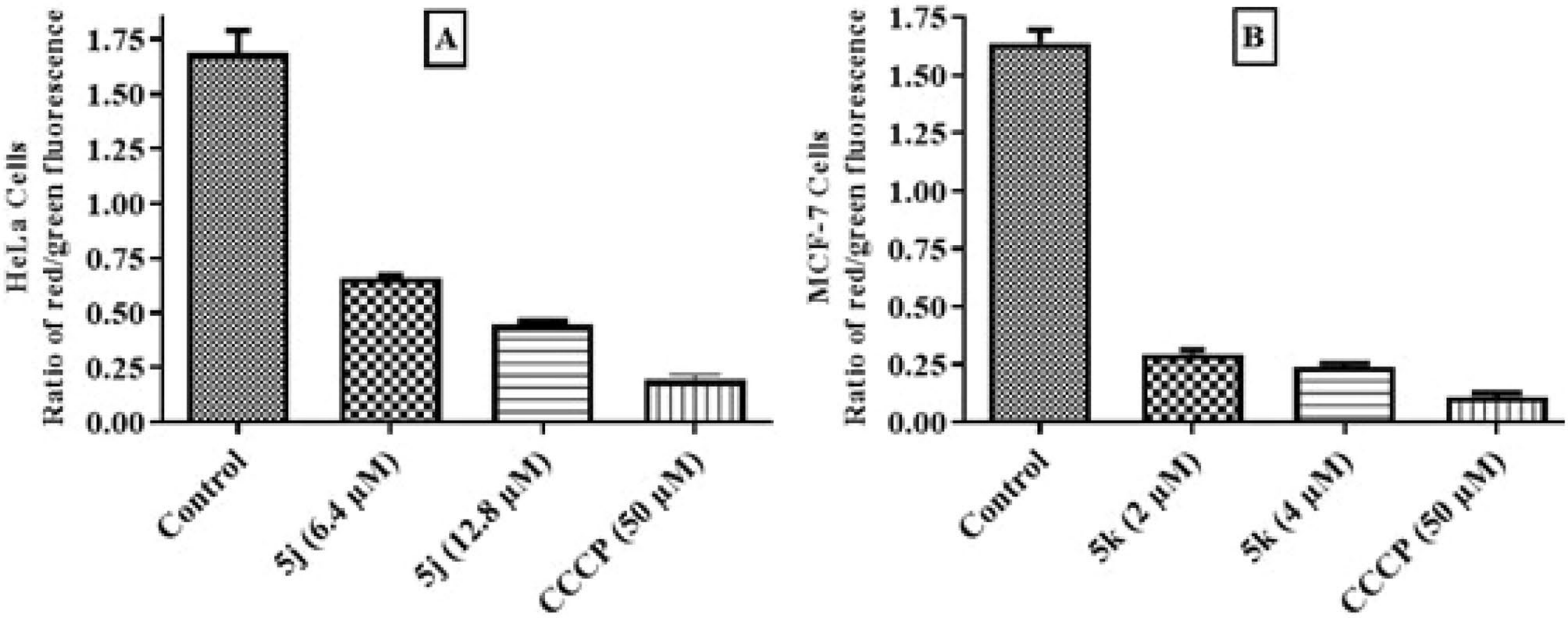
Quantitative analysis of depolarization of mitochondrial membrane potential in compounds **5j** and **5k** treated cells by observing ratio of red/green fluorescence after JC-1 staining. The untreated (control) cells maintained normal ΔΨm while treated cells are showing compromised ΔΨm. Data are represented as mean ± SD in replicate of three. *p ˂ 0.05, **p ˂ 0.01, ***p ˂ 0.001 versus control group (n = 3).

The cationic dye JC-1 was utilized to evaluate MMP in HeLa and MCF-7 cells after treatment with potent compounds **5j** and **5k** at their IC_50_ and 2 × IC_50_ values. A decrease in red/green ratio was observed in both HeLa and MCF-7 cells which indicated that the compounds caused a decrease in the mitochondrial membrane potential. The effect was comparatively less in HeLa cells as compared to MCF-7 cells as shown in Fig. 8.

### Determination of activated caspase-9 and caspase-3/7

**Figure 9:**
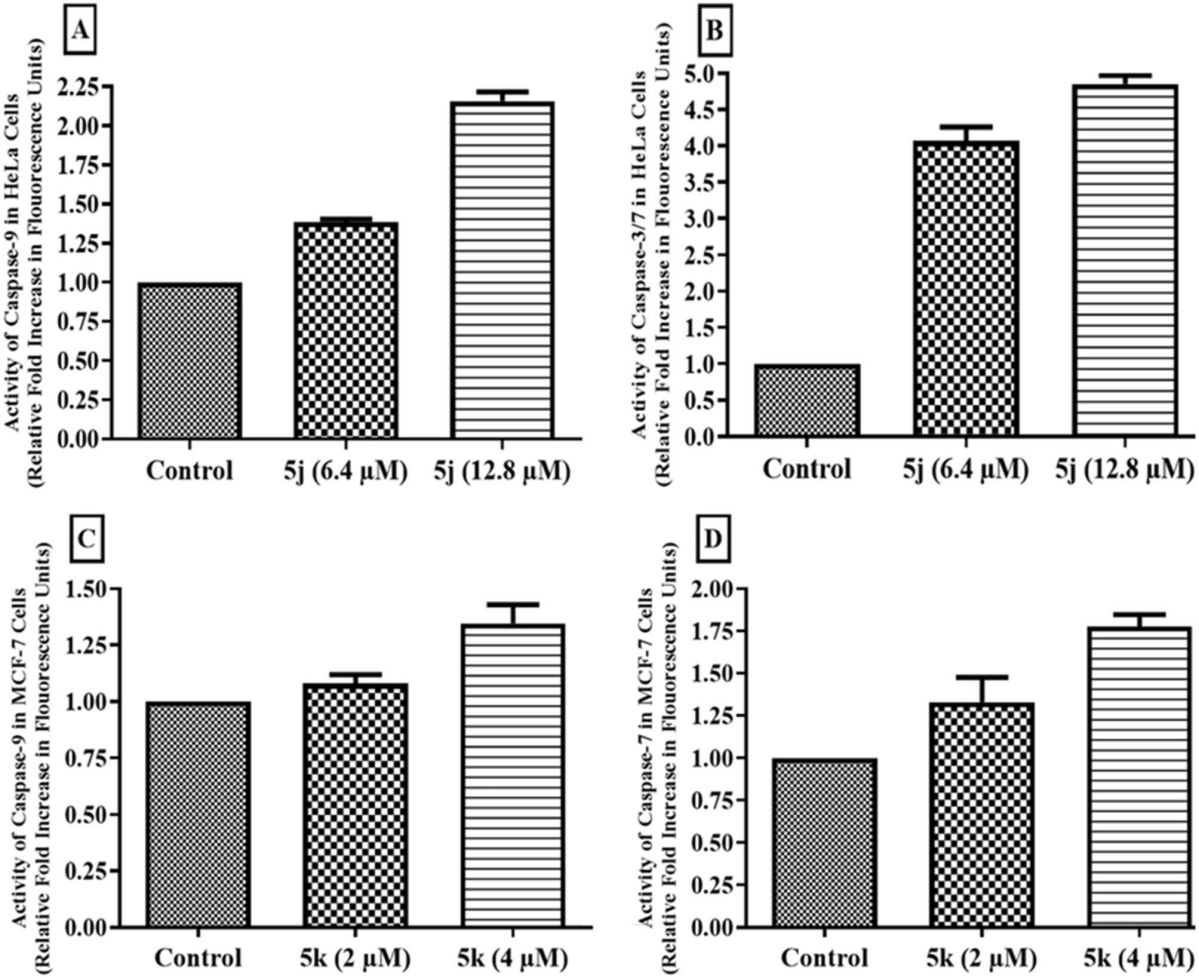
Fold increase in activity of (**A**) caspase-9 and (**B**) caspase-3/7 in HeLa cells after treatment with compound **5j.** Fold increase in (**C**) caspase-9 and (**D**) caspase-7 in MCF-7 cells after treatment with compound **5k**. Data are represented as mean ± SD in replicate of three. *p ˂ 0.05, **p ˂ 0.01, ***p ˂ 0.001 versus control group (n = 3).

The compounds **5j** and **5k** were selected for carrying out assays of caspase activation in HeLa and MCF-7 cells respectively. An increase of 1.4 and 2.2-folds was observed for activated caspase-9 while about 4 and 4.8-folds increase in activated caspase-3/7 was observed in HeLa cells treated at IC_50_ and 2 × IC_50_ values of compound **5j**, respectively. A fold increase of 1.1 and 1.4-folds in activated caspase-9 whereas, 1.4 and 1.8-fold increases in activated caspase-7 was observed in MCF-7 cells treated with compound **5k** at IC_50_ and 2 × IC_50_ values, respectively (Fig. 9).

## Discussion

The MTT assay is the most commonly used high throughput method to screen potential anti-proliferative compounds and is based on the reduction of a water soluble, yellow dye, MTT, to water insoluble, purple colored formazan crystals by the action of oxidoreductases present in healthy cells^31^. In present study, compounds **5j** and **5k** were found to be the most active compounds with IC_50_ values of 6.4 ± 0.45 and 2.03 ± 0.23 µM against HeLa and MCF-7 cells respectively. The structure activity relationship was determined based on the anti-proliferative effects observed in the MTT cell viability assay. In case of pyrazolopyridines (**4a**–**4n**), an H atom was present at position R_1_ (**4a**–**4g**). When an H atom was at R_1_, a mild increase in anti-proliferative activity was observed in HeLa cells. Only two compounds (**4c**, **4d**) showed good anticancer activity (i.e., having > 50% inhibition at 100 µM) when R_1_ was an H-atom and R was one of two strongly electronegative halogen atoms. When H atom at R_1_ was replaced by methyl group (**4h**–**4n**), a decrease in anti-proliferative activity was observed when there were either H or less bulky electronegative atoms like –F and –Cl groups at R_1_ (**4h, 4j** and **4k**). A mild increase in activity was observed when the position R was a –CH_3_ group (**4i**) or bulky electronegative atom or groups like –Br, –NO_2_, –OCF_3_ (**4l, 4m** and **4n**).

In case of pyrazolonaphthyridines (**5a**–**5n**), all compounds were found active except two compounds (**5f** and **5m**). The H atom was kept constant at R_1_ and substitutions were made at R (**5a**–**5g**). When the H atom was present at R (**5a**), an increase in anti-proliferative activity was noticed which further increased when H atom at R was replaced by –CH_3_ (**5b**) group. Substitution of –NO_2_ group (**5f**) yielded the least active compound which exhibited an inhibition of less than 50%. Bromo substitution (**5e**) yielded more active compound as compared to chloro (**5d**) substitution. However, replacing halogens with –NO_2_ (**5f**) group yielded least active compound among these derivatives against HeLa cells. Activity was increased when -NO_2_ was replaced by –OCF_3_ group (**5g**) in both cancer cell lines. Among seven derivatives (**5h**–**5n**), when R_1_ was a methyl group (-CH_3_), fluoro substitution at R yielded the most active compound (**5j**) in HeLa cells. Anti-proliferative activity was dependent on strength of electronegativity (**5j** ˃ **5k** ˃ **5l**) against HeLa cells. Substitution of –NO_2_ (**5m**) caused a decrease in activity. Substitution of –NO_2_ did not yield active compounds. The anti-proliferative potential of these compounds was attributed to the presence of halogen atoms at R position of benzene ring attached to naphthyridine ring.

Further mechanistic studies were designed to evaluate possible mechanism of action of the most active compounds at fairly effective doses. As the number of cells was high in these assays as compared to MTT assay so IC_50_ and 2 × IC_50_ values, calculated in MTT assay, were chosen in order to have a significant number of cells at the end of experiment against each dose. LDH assay is a reliable method to determine the cytotoxic potential of a test compound in cells. This assay is based on the fact that dying cells possess a highly permeable membrane as compared to live cells. Cellular contents can leak out of this permeable membrane. The LDH is a cytosolic enzyme which is responsible for interconversion of pyruvate and lactate during glycolysis^32,33^. At end of apoptosis, when cells have a permeable or ruptured membrane, the LDH that leaks out of cell is measured and the cytotoxicity of the test compound calculated with respect to positive and negative control. The treatment of cells with compounds **5j** and **5k** showed an increase in leaked LDH which depicted that these compounds have a sound cytotoxic potential against cancer cells.

Apoptotic and dying cells have a permeable cell membrane. Cell impermeable molecules can easily enter through permeable membranes. Fluorescence microscopy has made it possible to visualize apoptotic features in cells using fluorescence probes. Nuclear staining dyes propidium iodide (PI) and 4′,6-diamidino-2-phenylindole (DAPI) selectively enter in apoptotic cells and cross nuclear membrane where upon binding to DNA, their fluorescence increases manifold. Cell detachment, shrinkage of cells and condensation of nuclear material was increased in a dose dependent fashion after treatment with compounds **5j** and **5k** which depicted that potent compounds have potential to induce apoptosis in cancer cells.

Reactive oxygen species (ROS) were considered as byproducts of metabolism but with the passage of time their role in cell signaling was also discovered. They both are equally essential to healthy cells, as well as toxic, inducing different metabolic diseases and even apoptosis depending upon their level of production. At low levels, they are involved in cell signaling pathways while at higher levels, they are toxic to cells^34,35^. High oxidative stress triggers apoptosis in cancer cells^36,37^. A dose dependent ROS generation in treated cells may be attributed to dysfunctioning of mitochondria which may lead to apoptosis via a mitochondrial pathway^38^. Our results were in compliance with a published study^39^.

The cell cycle is a highly regulated and organized cellular process that occurs in parallel to apoptosis to maintain homeostasis. In cancer cells, the cell cycle process becomes continuous and uncontrolled due to mutational changes in regulating genes. Anticancer agents trap this cell cycle and can cause an arrest in this process at any stage, ultimately pushing these cells to apoptosis. Cyclin-CDK (cyclin dependent kinases) complexes are the main cell cycle regulatory proteins which may be down-regulated due to interactions with anti-proliferative agents. DNA content varies in different phases of the cell cycle which can be detected in the cells using fluorescent probes through flow cytometry. Compound **5j** showed G0/G1 arrest in HeLa cells that may be attributed to inhibition of cyclin D-CDK 4 and cyclin E-CDK 2 complex^40^.

Most anticancer agents target DNA and the interactions with DNA produce their apoptotic effect. The interaction of potent compound **5j** with DNA was studied: hypochromicity for two chromophores was observed with no red or blue shift, which indicated that compound **5j** was non-covalently bound to DNA. The docked conformation of **5j** against DNA exhibited an intercalation binding mode (as expected due to the rigid planar rings of the molecule). A hydrogen bond was observed between the ring oxygen atom of guanine nucleic acid base and the amino group. A number of hydrophobic pi-pi stacked interactions were observed between bases G and C with the aromatic rings of **5j**. The observed intercalative interaction was due to pi-pi stacked interactions of aromatic rings of **5j** with DNA bases and is in compliance with a published study^30^. Moreover, compound **5j** showed DNA damage in treated cells which revealed that the compound binds to DNA and causes DNA damage to induce apoptosis.

Mitochondria are important organelles in cells which manage cellular energy needs. Mitochondrial membrane has a relatively high negative charge as compared to cell membrane. Some apoptotic agents may cause damage to the mitochondrial membrane and consequently lead to a decrease in mitochondrial membrane potential (MMP). Increased mitochondrial membrane permeability leads to release of cytochrome c which combines with Apaf-1(apoptotic protease activating factor 1) to form apoptosome that consequently activate caspases which ultimately executes the process of apoptosis^41^. Compounds **5j** and **5k** caused a dose dependent decrease in MMP which was in compliance with a published study^42^.

Caspases are enzymes which are mostly activated in the apoptotic cascade. They are non-functional or downregulated in cancer cells due to mutational changes. These enzymes are proteolytic in their functionality and make the cells non-functional due to the loss of essential proteins during an apoptotic cascade. Caspase-9 is activated due to the formation of apoptosome which activates executioner caspases-3/7. Compounds **5j** and **5k** caused an increase in activated caspases in treated HeLa and MCF-7 cells respectively and confirming their apoptotic potential against cancer cells. Our findings were in compliance with a previous study^39^.

Pyrazolo pyridine derivatives (**4a**–**4n**) were found, in general, to be less active than cisplatin with only two active compounds (**4c** and **4d**) showing higher IC_50_ values than cisplatin. Pyrazolo-naphthyridines were highly active against cervical cancer (HeLa) and breast cancer (MCF-7) cells except compound **5f** and **5m**. Compounds **5j** and **5k** were the most active compounds inducing apoptosis in HeLa and MCF-7 cells at micromolar concentrations. The cytotoxic potential against cancer cells for both compounds was confirmed through LDH assay. A G0/G1 cell cycle arrest, higher cytotoxic and apoptotic potential observed through fluorescence microscopy using PI and DAPI staining, an increased oxidative stress confirmed by H_2_DCF-DA staining, intercalative binding with DNA and DNA damaging effects in treated cancer cells represented the pro-apoptotic activity of most active compounds. The potent compounds (**5j** and **5k**) also induced a decrease in mitochondrial membrane potential and an increase in activated caspases in treated HeLa and MCF-7 cells, both are indicative of the activation of the mitochondrial pathway of apoptosis induced by these compounds in cancer cells. Conclusively, the potent compounds (**5j** and **5k**) can be used as lead candidates for the development of drug against cervical and breast cancers.

## Materials and method

### Materials

All the chemicals used for the synthesis of naphthyridine derivatives, were purchased from Sigma-Aldrich. Lyophilized Herring sperm DNA, 3-(4,5-dimethylthiazolyl-2)-2,5-diphenyltetrazolium bromide (MTT, Lot# MKBQ2167V), propidium iodide (PI, Lot# SLBH8362V), 4′,6-diamidino-2-phenylindole (DAPI, Lot# 034M4030V), 2′,7′-dichlorodihydrofluorescein diacetate (H_2_DCF-DA) and 5′,5′,6′,6′-tetrachloro-1′,1′,3′,3′-iodide (JC-1, Lot# MKBR2378V) dyes, agarose (Low gelling temp.).

CCCP (Carbonyl cyanide *m*-chlorophenyl hydrazone) were purchased from Sigma Aldrich, USA. Cell culture components RPMI (Rosewell Park Memorial Institute Medium, Lot # 688600), DMEM (Dulbecco’s modified Eagles Medium, Lot# 02150011), fetal bovine serum (FBS, Lot # 2440071), trypsin–EDTA (Lot# 2259320) and penicillin streptomycin (Pen/Strep, Lot# 2321148) were purchased from Gibco, USA. Phosphate buffered saline (PBS) tablets, RNase A, and agarose from Invitrogen, USA, Triton X-100 from AppliChem, Germany and formaldehyde (37%) from Scharlau, Spain, H_2_O_2_ (Hydrogen Peroxide) (30%) from Thermo-Fisher Scientific, dimethyl sulfoxide DMSO (sterile) from DAEJUNG, Korea, Albumin Fraction V (Bovine Serum Albumin, BSA) from Carl Roth Gmbh & Co. Kg, Germany were also used in biological assays.

Breast cancer (MCF-7, ATCC^®^ HTB-22™), cervical cancer (HeLa, ATCC^®^ CCL-2™) and Baby Hamster kidney (BHK-21, ATCC^®^ CCL-10™) cells were gifted by Dr. Syed Shahzad ul Hussan from Lahore University of Management Sciences (LUMS), Lahore, Pakistan. Main stocks of these cells were cryopreserved at -196 °C. MCF-7 cells were grown in DMEM supplemented with 15% FBS and 1% Pen/Strep while HeLa and BHK-21 cells were grown in RPMI supplemented with 10% FBS and 1% Pen/Strep.

### Synthesis of synthesis of pyrazolo-pyridine (**4a**–**4n**) and pyrazolo-naphthyridine (**5a**–**5n**) derivatives

**Scheme 1:**
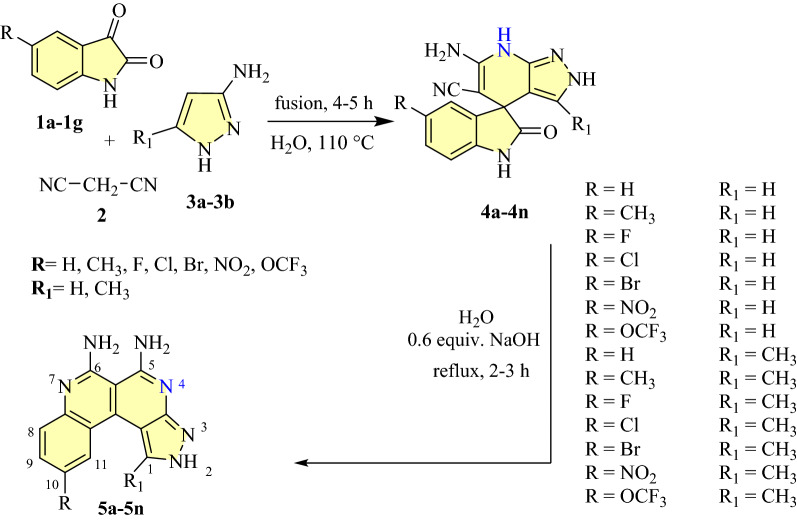
Preparation of pyrazolo-pyridine (**4a**–**4n**) derivatives and pyrazolo-naphthyridine (**5a**–**5n**) derivatives^43^.

The synthesis of spiro pyrazolo-pyridine (**4a**–**4n**) and pyrazolo-naphthyridine (**5a**–**5n**) derivatives were carried out by following the Scheme 1 as reported previously^43^.

### MTT cell viability assay

The anti-proliferative activity of the studied compounds was determined against breast (MCF-7) and cervical (HeLa) cancer cells by the MTT cell viability assay as described earlier^44,45^. BHK-21 cells were used as non-cancerous cells to study the effect of these compounds on normal cells. Briefly, 1 × 10^4^ cells/well were seeded in 96-well microtiter plate and placed in an incubator (Heracell™ VIOS 160i, Thermo-Fisher Scientific) set for 5% CO_2_ at 37 °C. The stock solutions of compounds were prepared at 10 mM concentration in DMSO. Initial working solutions were prepared in cell culture medium at 1 mM concentration. Compounds exhibiting more than 50% inhibition at 100 µM end concentration were further tested at lower concentrations (100 to 2.5 µM) to calculate IC_50_ values. After 24 h, medium in each well was aspirated and cells were treated for 4 h with serum free medium containing 0.2 mg/mL MTT reagent. The solubilizing reagent (10% acidified sodium dodecyl sulfate in propanol in a 1:1 ratio) was added to solubilize formazan crystals. Plates were kept at 37 °C for 30 min and then placed on a gyratory shaker for few min for complete solubilization of formazan crystals. The optical density was measured at 570 nm by microplate reader and IC_50_ values were calculated from three independent experiments as previously reported^46^.

### LDH cytotoxicity assay

The cytotoxic potential of the most active compounds was evaluated by measuring the lactate dehydrogenase released into cell culture medium by compound-treated cells using the Pierce LDH Cytotoxicity Assay Kit (Lot# SF252000, Thermo Scientific). Briefly, 1 × 10^4^ cells/well were seeded in a flat bottom 96 well cell culture plate and kept for overnight incubation. Cells were treated with potent compounds **5j** and **5k** at the IC_50_ and 2 × IC_50_ values. The plate was centrifuged at 1500 rpm for 3 min and the supernatant (50 µL) transferred to a new sterile 96-well plate. After addition of substrate mix (50 µL), plate was placed at room temperature for 30 min. The absorbance was measured at 490 nm and 680 nm using microplate reader (FLUOstar Omega, Ortenberg, Germany). Cytotoxicity was measured as described earlier^47^.

### Microscopic analysis of apoptosis

Morphological changes in compound treated cancer cells were identified using propidium iodide (PI) and 4′,6-diamidino-2-phenylindole (DAPI) staining with observing under a fluorescence microscope (Nikon ECLIPSE Ni–U, Japan). Briefly, 2 × 10^5^ cells/well were grown on microscopic slides and treated with IC_50_ and 2 × IC_50_ values of the most active compounds **5j** and **5k**. After 24 h, media was aspirated, cells were washed with sterile phosphate buffered saline (PBS) and fixed by using 4% formalin and 0.1% Triton X–100 as adherent cells fixation solution. Further, 10 µL (0.01 mg/mL) of PI or DAPI solution was added on slides and kept for 10 min in dark. Fluorescence photomicrographs were captured as previously reported^48^.

### Determination of oxidative stress

The potential of most active compound to induce apoptosis in treated cancer cells via production of reactive oxygen species was analyzed by fluorescence microscope (Nikon ECLIPSE Ni–U, Japan) after 2′,7′-dichlorodihydrofluorescein diacetate (H_2_DCF-DA) staining. Cells (2 × 10^5^) grown on microscopic slides were treated with IC_50_ and 2 × IC_50_ values of the most active compounds **5j** and **5k**. After washing with sterile PBS, cells were fixed and 10 µL (0.01 mg/mL) of H_2_DCF-DA dye was added on microscopic slides. After an incubation of 10 min at room temperature in the dark, fluorescence photomicrographs were obtained as previously reported^49^.

### Cell cycle analysis assay

The variation in cell cycle distribution was observed in cancer cells treated with potent compound **5j** by flow cytometer (BD Accuri™ C6, USA). Briefly, HeLa cells (2 × 10^5^ cells/mL) were grown and treated with IC_50_ and 2 × IC_50_ values of potent compound **5j**. Cells were harvested and washed three times with PBS to remove traces of cell culture medium. Cells were fixed in 70% ethanol for 24 h at − 20 °C. Pelletized cells were then re-suspended in 1 mL of a solution containing PI (20 µg/mL), RNase A (50 µg/mL) and 0.1% (v/v) Triton X-100. Samples were kept in the dark for 30 min at room temperature and analyzed within an hour as reported earlier^50^.

### DNA binding studies

The interaction of the most active compound **5j** with herring sperm DNA was performed following a published methodology^48,51^. A stock solution of 5 mg of DNA was prepared in 10 mL of distilled water and the purity of the DNA (A_260_/A_280_) was in the range of 1.6 to 1.9. Compound **5j** was tested at a final concentration of 200 µM with varying end concentrations (0 to 396 µM) of DNA. The plate was placed at room temperature for 30 min and the UV absorption spectrum was recorded by microplate reader (FLUOstar Omega BMG Labtech, Ortenberg, Germany).

### DNA docking studies

For DNA-docking studies, the crystal structure of DNA was downloaded from the PDB (PDB id: 1kci). AutoDock Vina was used for the docking studies. The docking method was first validated by re-docking the co-crystallized ligand, the software was able to give the same conformation (< 2 Å rmsd) of the ligand as seen in the crystal structure.

### Comet assay

Comet assay is a sensitive method to determine DNA damage which was performed after some modifications in a published protocol^52^. DNA binding and molecular docking studies suggested intercalation of compound with DNA, so a single dose (IC_50_ value) was sufficient to validate these findings by evaluating DNA damage through comet assay. The cervical cancer (HeLa) cells were treated with IC_50_ value of potent compound **5j** for 24 h. Cells were harvested, collected and centrifuged to obtain a pellet. Pelleted cells were re-suspended in media. Untreated cells were used as control cells. For the positive control, 2 × 10^4^ cells were treated with 20 µM of freshly prepared hydrogen peroxide for 30 min. Slides were prepared by coating with 1% normal melting point agarose (NMPA) on one side and then dried. Samples (2 × 10^4^ cells/sample) were re-suspended in 1 mL of PBS. In a tube, 0.4 mL cell suspension was mixed with 1.2 mL of 0.9% low melting point agarose (LMPA) and mixed thoroughly by pipetting. A cell suspension of 65 µL was laid on coated slide and evenly distributed by placing a coverslip over it. Slides were placed at − 20 °C for 10 min. Then the slides were placed at 4 °C in the dark in a lysis buffer of 100 mM EDTA, 10 mM Trizma base, 2.5 M sodium chloride, 10% DMSO and 1% Triton X-100 in distilled water with a pH of 10. After an overnight incubation, slides were washed with chilled distilled water. These slides were then placed in electrophoresis chamber (Wealtec, USA) having chilled (4 °C) alkali unwinding solution (300 mM sodium hydroxide and 1 mM EDTA in distilled water). Electrophoresis was carried out at an adjusted voltage (between 0.7 and 1.0 V/cm) for 35 min in an electrophoresis tank. Slides were then immersed in neutralization solution (0.4 M Trizma base in 800 mL water, pH 7.5) for 10 min in dark. Propidium iodide (20 µL of 0.01 mg/mL) was used as fluorescent dye to stain DNA. Slides were placed in dark at room temperature. After 10 min, images were captured using a fluorescence microscope (Nikon ECLIPSE Ni–U, Japan) at an excitation/emission wavelength of 493/632 nm. Microscopic images were analyzed using ImageJ (ij152-win-java8, National Institutes of Health, Bethesda, USA) and OpenComet (OpenComet_imagej_v1.3.1, USA) where the DNA content in the head and tail of the comets were automatically measured by the software. Results of three independent experiments were expressed as tail length (µM), %DNA in comet tail and comet tail moment.

### Measurement of mitochondrial membrane potential (ΔΨm)

Mitochondrial membrane potential was determined by a fluorimetric method using fluorescent dye 5′,5′,6′,6′-tetrachloro-1′,1′,3′,3′-iodide (JC-1). Briefly, 1 × 10^5^ cells were grown and treated with IC_50_ and 2 × IC_50_ values of compounds **5j** and **5k** for 24 h. Cells were harvested, pelletized and treated with 0.25 µM of fluorescent dye JC-1. Each sample was transferred to a flat-bottomed black 96-well plate, which was kept in the dark for 20 min. Fluorescence emission was measured by a fluorescent microplate reader (FLUOstar Omega BMG Labtech, Germany) at 590 nm and 520 nm for j-aggregates and j-monomers. Results were measured as ratio of red/green fluorescence as previously reported^53^.

### Apoptosis assessment by caspase -9 and -3/7 activity

Activation of apoptosis inducing caspases were determined using fluorimetric assay kits by Abcam (ab65607 (Lot# GR3194417-1) & ab39383 (Lot# GR3188949-1). Briefly, 1 × 10^6^ cells were grown and treated with IC_50_ and 2 × IC_50_ values of potent compounds **5j** and **5k**. Cells were harvested after 18 h’ treatment and washed with PBS. Pelletized cells were treated with lysis solution and the lysate transferred to new eppendorf tubes. After protein quantification using “Total Protein Biuret (Lot# 210-B) Kit”, the lysate was treated with substrates of caspase-9 and caspase-3/7 in a black 96-well plate. Fluorescence excitation/emission at 400/505 nm was measured by fluorescence microplate reader (Bio–TeK FLx800™, Instrument, Inc. USA). Results were represented as fold increase in activated caspase-9 and caspase-3/7 as compared to control as previously reported^46^.

### Statistical analysis

The data was presented as the mean ± SEM or mean ± SD of at least three independent experiments. Statistical analyses were performed using Student’s t-test and graphs were drawn by GraphPad Prism (version 8.0.2). Values of p ≤ 0.05 were considered statistically significant.

## Data Availability

The datasets used and/or analysed during the current study available from the corresponding author on reasonable request.
